# Retrospective analysis of non-invasive prenatal testing: a population study involving 19,835 participants in the Shaoyang area

**DOI:** 10.3389/fgene.2025.1748107

**Published:** 2026-01-09

**Authors:** Muping Zhou, Hua Zhang, Jun Wen, Na Liu, Zhongchun Wu, Ting Lin, Mengyun Tang, Yan Huang, Ying Xiao, Hui Xie, Xia Ouyang, Xiang Xiao, Liyuan Deng, Ning Chen, Ani Zeng, Jinping Peng

**Affiliations:** 1 Shaoyang Prenatal Diagnosis Center, The Maternal and Child Health Hospital of Shaoyang City, Shaoyang, China; 2 Department of Pathology, The Central Hospital of Shaoyang, Shaoyang, China

**Keywords:** NIPT, parental ages, PPV, prenatal diagnosis, sex chromosome abnormalities, trisomy

## Abstract

**Background:**

This cohort study aimed to evaluate the clinical efficacy of Non-invasive prenatal testing (NIPT) in detecting fetal chromosomal abnormalities within a pregnant population in the Shaoyang area, and to further investigate the potential correlation between parental ages and the risk of such abnormalities.

**Methods:**

A cohort study was conducted involving 19,835 pregnant women recruited for NIPT between October 2019 and April 2024. The performance of NIPT was assessed based on its positive predictive value (PPV), sensitivity, and specificity. The relationship between parental age and chromosomal abnormalities was analyzed.

**Results:**

We identified 88 positive cases, among which Trisomy 13, 18, and 21 accounted for 64 cases. The total PPV was 75.29%. Additionally, we detected 28 cases of sex chromosomal aneuploidies, with an overall PPV of 24.56%. The detection rate of autosomal abnormalities was higher in parents aged 35 years or older (5.49‰) compared to that in the younger parental age group (2.62‰). Unexpectedly, the sex chromosomal abnormalities exhibited an opposite trend, with the younger group demonstrating a higher detection rate of 1.31‰ than the advanced parental age group (0.61‰). All 7 cases of 47,XXY were concentrated among parents under 35 years of age. These results indicate that advanced parental ages are associated with an increased risk of autosome abnormalities; conversely, younger parental ages appear to correlate with a heightened risk of sex chromosome abnormalities.

**Conclusion:**

Parental ages may thus influence the occurrence of chromosome abnormalities. Our findings provide novel insights for prenatal genetic counseling.

## 1 Introduction

Fetal chromosomal abnormalities (including aneuploidy, copy number variations [CNVs], and mosaicism) represent a fundamental cause of birth defects. These abnormalities interfere with critical pathways in embryonic development. This disruption can lead to complex clinical phenotypes characterized by multi-organ system malformations, cognitive function deficits, gonadal developmental anomalies, and somatic growth disorders, and can even pose a threat to life (Zhou et al., 2024; Lv et al., 2021). These conditions frequently exhibit irreversibility and currently lack effective treatment options, thereby imposing a significant burden on both families’ quality of life and public health resources. In this context, enhancing prenatal screening technologies and diagnostic strategies is essential for interrupting the cycle of births involving chromosomally abnormal fetuses and improving population quality (Laforce et al., 2025; Heinke et al., 2021).

Non-Invasive Prenatal Testing (NIPT) has emerged as an innovative screening technique that analyzes fetal cell-free DNA from maternal peripheral blood. Utilizing high-throughput sequencing technology for detecting chromosome aneuploidies, NIPT offers several advantages: it is non-invasive, safe, and highly accurate. Consequently, it has become an important method for clinical screening of common fetal chromosome aneuploidies such as trisomy 13, 18, and 21 (T13/T18/T21) (Jayashankar et al., 2023). However, this technique can be influenced by factors such as low fetal fraction in maternal plasma, placental mosaicism, and maternal chromosomal variations. It carries a certain risk of false positives (Zhang et al., 2024). Therefore, further confirmatory diagnosis via invasive prenatal diagnostic procedures is necessary for pregnant women identified as high-risk by NIPT (Di Renzo et al., 2023). Amniocentesis with karyotype analysis serves as the gold standard for diagnosing chromosomal abnormalities (Zheng et al., 2024); the combined application of cytogenetic and molecular genetic technologies allows precise identification of fetal chromosome number and structural anomalies - providing scientific evidence crucial for preventing birth defects. Given this context, we analyzed data from 19,835 NIPT cases with corresponding diagnostic results to evaluate the performance of the BGI platform. Additionally, we examined the correlation between parental age and the risk of chromosome abnormalities in offspring. Our study may represent the first report utilizing NIPT data to investigate the relationship between parental age and the risk of chromosomal abnormalities in offspring, thereby providing a novel perspective for assessing and preventing risk factors associated with chromosomal abnormalities.

## 2 Materials and methods

### 2.1 Subjects

**FIGURE 1:**
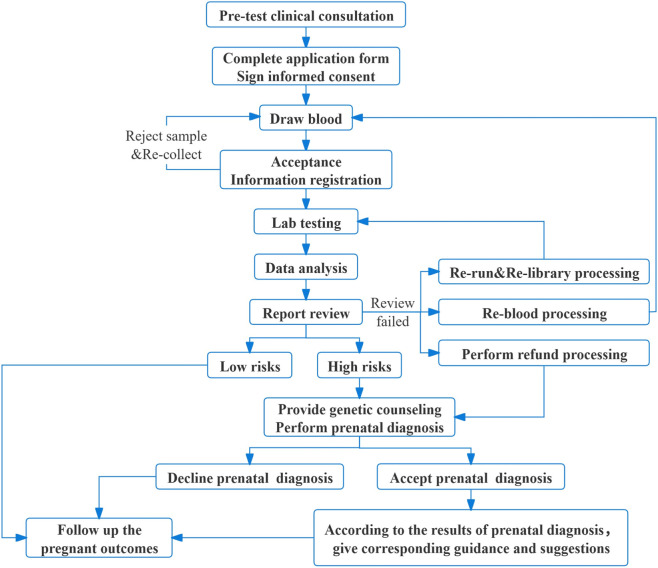
The workflow of NIPT for detecting chromosomal abnormalities.

The study enrolled 19,835 pregnant women from Shaoyang who voluntarily underwent NIPT and met the eligibility criteria between 1 October 2019, and 30 April 2024. All participants were fully informed about the necessity and risks of prenatal screening/diagnosis and provided written informed consent. The study protocol is summarized in Figure 1. Ethical approval (SYMCHH-EC-2024-001) was obtained from the Ethics Committee of Maternal and Child Health Hospital of Shaoyang City, and written informed consent was obtained from all participants before enrollment.

### 2.2 BGI sequencing method

Peripheral blood samples (5 mL) from pregnant women were collected in EDTA anticoagulant tubes and stored at 4 °C, followed by double centrifugation within 8 h. Cell-free DNA from plasma was extracted using a DNA extraction kit produced by Shenzhen BGI Genomics Co., Ltd. The extracted cell-free DNA underwent library construction for the detection of fetal chromosomal abnormalities using a detection kit provided by BGI Biotechnology (Wuhan) Co., Ltd. The resulting libraries were sequenced on the BGISEQ-500 platform developed by Shenzhen BGI Intelligent Manufacturing Technology Co., Ltd., utilizing high-throughput sequencing technology. Raw data were aligned with the human genome reference sequence (GRCh37/hg19), and Z-tests were employed to assess whether there were significant differences between the test samples and normal controls, thereby determining the risk of chromosomal abnormalities.

### 2.3 Invasive prenatal diagnosis

Patients with high-risk NIPT results received genetic counseling and were referred for invasive prenatal diagnosis. Amniocentesis was then performed under ultrasound guidance, collecting 20 mL of amniotic fluid for karyotyping and chromosome microarray analysis (CMA).

### 2.4 Analysis of CMA

The genomic DNA was extracted using a extraction kit produced by QIAGEN, Germany. The samples from amniotic fluid were analyzed using the CytoScan 750K chip (550,000 CNV probes +200,000 SNP probes), which focuses on prenatal applications; all raw data obtained were analyzed with Affymetrix Chromosome Analysis Suite Software. To assess the pathogenicity of CNVs, multiple authoritative databases such as Decipher, UCSC Genome Browser, OMIM (Online Mendelian Inheritance in Man), ISCA (International Standards for Cytogenomic Arrays), and DGV (Database of Genomic Variants) were referenced alongside relevant literature.

### 2.5 Statistical analysis

The objective of this study is to analyze the number of NIPT tests conducted from 1 October 2019, to 30 April 2024, as well as the count of high-risk cases. Additionally, we will assess the instances of true positives, false positives, true negatives, and false negatives within this dataset.

Several key metrics were calculated in our study:

Detection rate=true positives / total tests×100%

Positive rate=high−risk cases / total tests×100%

$$\text{Positive predictive value }\left(\text{PPV}\right)=\text{true positives }/\;\left(\text{true positives}+\text{false positives}\right)\times 100\%$$

$$\text{Sensitivity}=\text{true positives }/\;\left(\text{true positives}+\text{false negatives}\right)\times 100\%$$

$$\text{Specificity}=\text{true negatives }/\;\left(\text{true negatives}+\text{false positives}\right)\times 100\%$$

All statistical analyses and visualizations were performed using R software (version 4.5.1). The following packages were employed: the “openxlsx” package (version 4.2.8.1) for data import; the “ggplot2” package (version 4.0.1) for creating graphs; the “ggpubr” package (version 0.6.2) for generating publication-ready plots and for statistical annotations; the “ggpmisc” package (version 0.6.3) for adding statistical details to plots; and the “ggExtra” package (version 0.11.0) for adding marginal distributions. A 95% confidence interval (CI) was calculated. Chi-square tests and t-tests were conducted to assess statistical significance, with a p-value of <0.05 considered statistically significant.

## 3 Results

### 3.1 Positive results of NIPT

We conducted 19,835 NIPT tests, of which 236 were positive. The most frequently detected trisomies were T13, T18, and T21, with 103 cases (43.10%): 66 cases of T21, 21 of T18, and 16 of T13. Additionally, we identified 13 types of rare autosomal trisomies (RATs), totaling 30 positive findings (12.55%). Sex chromosome aneuploidies accounted for 63 cases (26.36%), including 37 cases of 45,X, 14 of 47,XXY, 9 of 47,XYY, and 3 of 47,XXX. For CNVs, NIPT detected 43 positive cases (17.99%), comprising 12 autosomal microdeletions, 9 autosomal microduplications, 16 X-chromosomal microdeletions, and 6 X-chromosomal microduplications. The comprehensive results are presented in Table 1.

**TABLE 1:** Classification of 19,835 cases of NIPT positive results.

Classification	Counting	Proportion
T13/T18/T21	103	43.10%
T13	16	6.69%
T18	21	8.79%
T21	66	27.62%
RATs	30	12.55%
T2	1	0.42%
T3	4	1.67%
T4	1	0.42%
T5	1	0.42%
T7	2	0.84%
T8	2	0.84%
T9	1	0.42%
T10	4	1.67%
T11	1	0.42%
T15	2	0.84%
T16	6	2.51%
T20	2	0.84%
T22	3	1.26%
SCAs	63	36.02%
45,X	37	15.48%
47,XXY	14	5.86%
47,XYY	9	3.77%
47,XXX	3	1.26%
CNVs	43	17.99%
Del	12	5.02%
Dup	9	3.77%
X del	16	6.69%
X dup	6	2.51%

Abbreviation: CNVs, copy number variations; Del, microdeletion; Dup, microduplication; RATs, rare autosomal aneuploidies; SCAs, sex chromosome abnormalities; T13/18/21, trisomies 13, 18 and 21; among other abbreviations, trisomy is abbreviated to T and no more redundant description.

### 3.2 The prenatal diagnostic outcomes

**FIGURE 2:**
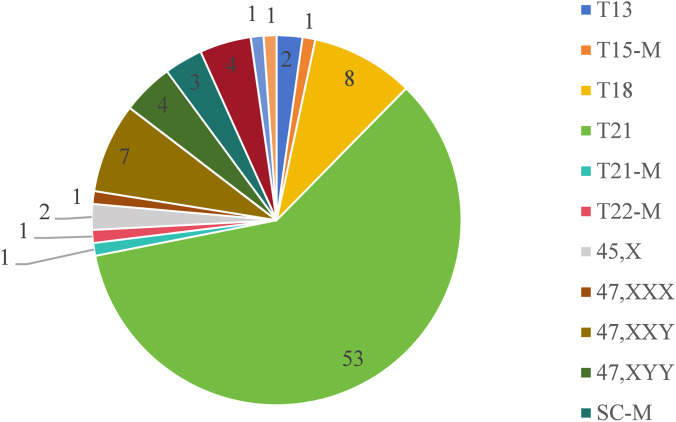
All positive cases diagnosed in our research. Legend: Abbreviation: CNV, copy number variation; CNV-M, copy number variation mosaicism; SC-CNV, sex chromosome copy number variation; SC-M, sex chromosome mosaicism; T13, trisomy 13; T15-M, trisomy 15 mosaicism; among other abbreviations, trisomy is abbreviated to T and no more redundant description.

n our study, we identified 236 cases with positive NIPT results. Among these, 35 cases were lost to follow-up, while 201 underwent prenatal diagnosis, confirming 88 true positives. The detected conditions included T21 in 53 cases, T18 in 8, T13 in 2, 45,X in 2, 47,XXX in 1, 47,XXY in 7, 47,XYY in 4, and CNVs in 4. Notably, one case involved a sex chromosome CNV (SC-CNV). Additionally, we detected mosaicism in 7 cases: one case each of T15 mosaicism (T15-M), T21 mosaicism (T21-M), and T22 mosaicism (T22-M) alongside three instances of sex chromosome mosaicism (SC-M). Furthermore, there was one case of CNV mosaicism (CNV-M). All confirmed results are summarized in Figure 2.

### 3.3 The performance analysis

We conducted a statistical analysis of the diagnostic results from all confirmed cases and calculated the PPV, sensitivity, and specificity for both autosome and sex chromosomal abnormalities (Table 2). The results indicate that the overall PPV for common trisomies (T13/T18/T21) was 75.29%, with the highest PPV observed for T21 (91.53%), while the lowest PPV for T13 (14.29%). Other autosome abnormalities (OAAs) had an overall PPV of 14.63%. The RATs have a PPV of 9.09%, while the PPV for CNVs in the autosomes was 20.00%. The combined PPV for all autosome abnormalities was 55.55%. A total of 18 cases of sex chromosomal abnormalities (SCAs) were diagnosed, yielding an overall PPV of 21.95%. Among these, the PPV for sex chromosome aneuploidies was 24.56%. The highest PPVs were observed for 47,XXX and 47,XXY, both at 50%, and the PPV for 47,XYY was 40.00%. Conversely, The lowest PPVs were for 45, X (6.67%) and SC-CNVs (4.55%). All sensitivity and specificity results are shown in Table 2.

**TABLE 2:** Analysis of PPV in chromosome abnormalities.

Abnormal types		Confirmed	Negative	Numbers	PPV	Sensitivity	Specificity
					%	%(95%CI)	%(95%CI)
T13/T18/T21	T13	2	12	14	14.29%	100.00 (34.24–100.00)	99.94 (99.86–99.98)
	T18	8	4	12	66.67%	100.00 (67.56–100.00)	99.98 (99.93–99.99)
	T21	54	5	59	91.53%	100.00 (93.07–100.00)	99.97 (99.93–99.99)
	Total	64	21	85	75.29%	100.00 (95.22–100.00)	99.89 (99.75–99.98)
Other autosomes	RATs	2	20	22	9.09%	66.67 (20.78–93.85)	99.90 (99.81–99.95)
	CNV	4	16	20	20.00%	80.00 (37.55–96.38)	99.92 (99.84–99.96)
	OAAs	6	35	41	14.63%	75.00 (30.06–95.44)	99.82 (99.70–99.89)
Total autosome abnormalities		70	56	126	55.55%	97.22 (90.33–99.33)	99.71 (99.55–99.82)
Sex chromosomes	45,X	2	29	31	6.67%	100.00 (34.24–100.00)	99.85 (99.75–99.92)
	47,XXX	1	1	2	50.00%	100.00 (20.65–100.00)	99.99 (99.96–100.00)
	47,XXY	7	7	14	50.00%	87.50 (52.91–97.76)	99.96 (99.91–99.98)
	47,XYY	4	6	10	40.00%	66.67 (24.46–94.00)	99.96 (99.91–99.98)
	Sex chromosome aneuploidies	14	43	57	24.56%	82.35 (59.75–93.64)	99.78 (99.64–99.87)
	CNV	1	21	22	4.55%	100.00 (20.65–100.00)	99.89 (99.75–99.95)
	Mosaicism	3	—	—	—	—	—
Total SCAs		18	64	82	21.95%	85.71 (64.55–95.21)	99.68 (99.53–99.78)

Abbreviation: CI, confidence interval; CNVs, copy number variations; OAAs, other autosome abnormalities; RATs, rare autosome aneuploidies; SCAs, sex chromosome abnormalities; T13, trisomy 13; among other abbreviations, trisomy is abbreviated to T and no more redundant description.

### 3.4 The relationship parental ages and chromosomal abnormalities

We stratified parental age into groups and analyzed the relationship between age and the PPV of chromosomal abnormalities. When maternal age is <35 years, the PPV for autosomal abnormalities (AAs) was 54.67%, while that for SCAs was 27.78%. In contrast, when maternal age is ≥35 years, the PPV for autosome abnormalities is 56.00%, whereas that for SCAs decreases to 12.50%. For paternal age <35 years, the PPVs for autosome and SCAs are 46.55% and 25.49%, respectively. Conversely, when paternal age is ≥35 years, these values rise to 63.24% and drop to 18.52%. When analyzing both parental ages together, it can be observed that when both parents are <35 years old, their respective PPVs are 47.27% and 26.53%. In contrast, if the parents are aged ≥35 years, these PPV shift to 57.45% and 13.64%. All the data are presented in Table 3. It can be concluded that when paternal age is ≥35 years, the PPV for AAs exceeds that of fathers <35 years; however, this trend reverses for SCAs where younger fathers exhibit a higher PPV. Similarly, couples with both partners under the age of 35 demonstrate lower rates of autosomal abnormality PPVs alongside higher rates of SCA PPVs.

**TABLE 3:** PPV of chromosomal abnormalities by age group.

Age types		Autosomal abnormalities		PPV	SCAs		PPV
		Confirmed	Negative		Confirmed	Negative	
MA	<35	41	34	54.67%	15	39	27.78%
	≥35	28	22	56.00%	3	21	12.50%
PA	<35	27	31	46.55%	13	38	25.49%
	≥35	43	25	63.24%	5	22	18.52%
PA < 35 & MA < 35		26	29	47.27%	13	36	26.53%
PA ≥ 35 & MA ≥ 35		27	20	57.45%	3	19	13.64%

Abbreviation: MA, maternal age; PA, paternal age; PPV, positive predictive value; SCAs, sex chromosome abnormalities.

**FIGURE 3:**
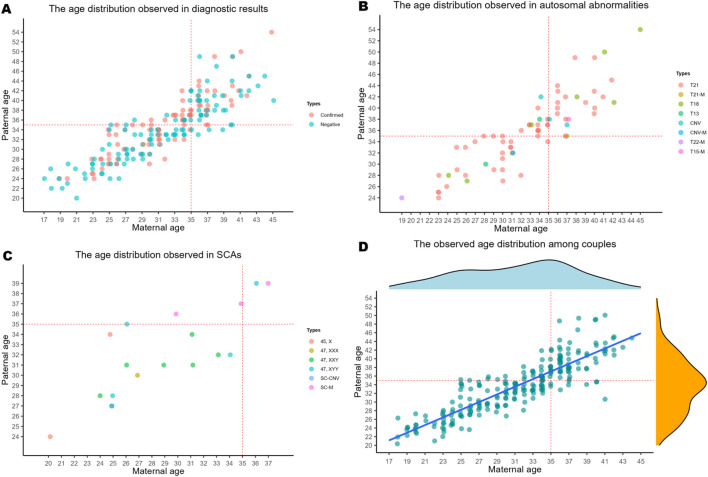
The correlation between parental age and chromosomal abnormalities in positive cases. Legend: The correlation between parental age and chromosomal abnormalities in positive cases. **(A)** The parental ages distribution observed in diagnostic results. **(B)** The parental ages distribution observed in autosomal abnormalities. **(C)** The parental ages distribution observed in SCAs. **(D)** The age distribution observed in couples. The Linear regression equation: y = 0.88 × x + 6.10, *r* ^2^ = 0.77; y means paternal age, x means maternal age.

In Table 4 and Figure 3A, we present an analysis of the distribution of parental ages across 201 diagnostic samples. Our findings reveal that when both parents are aged ≥35 years, there is a total of 69 samples, with 30 testing positive, accounting for 43.48% in this age group. Among the positive cases (Figure 3B), we identified 19 instances of T21, 5 instances of T18, 1 instance of T15-M, 2 instances of CNV, 1 instance of 47,XYY, and 2 instances of SC-M. When the paternal age is ≥35 years and the maternal age is <35 years, we observed a total of 25 samples; among them, there were 18 positive results, yielding a proportion of 72%. This includes 13 cases of T21 and one case each of T18, T13, CNV, 47,XYY, and SC-M. In contrast, when the paternal age is <35 years and the maternal age is ≥35 years, we identified only 5 samples with just one confirmed case (T21), leading to a proportion of 20.00%. Furthermore, there were 102 samples when both parents were under the age of 35 years. Thirty-nine cases were confirmed, resulting in a proportion of 38.24%. The breakdown is as follows: 21 cases of T21, 2 cases of T18, 45,X and 47,XYY, along with one case each of T13, T21-M, T22-M, 47,XXX, and SC-CNV. Finally, a total of 7 cases of 47,XXY were identified.

**TABLE 4:** Analysis of parental age in confirmed cases.

Chromosomal abnormalities	Parental age				
	PA ≥ 35 & MA ≥ 35	PA ≥ 35 & MA < 35	PA < 35 & MA ≥ 35	PA < 35 & MA < 35	Total
T13	0	1	0	1	2
T18	5	1	0	2	8
T21	19	13	1	20	53
T15-M	1	0	0	0	1
T21-M	0	0	0	1	1
T22-M	0	0	0	1	1
CNV	2	1	0	0	3
CNV-M	0	0	0	1	1
45,X	0	0	0	2	2
47,XXX	0	0	0	1	1
47,XXY	0	0	0	7	7
47,XYY	1	1	0	2	4
SC-M	2	1	0	0	3
SC-CNV	0	0	0	1	1
Total comfirmed	30	18	1	39	88
Negative sample	39	7	4	63	113
Proportion of AAs comfirmed	39.13%	64.00%	20.00%	26.26%	34.83%
Proportion of SCAs	4.35%	8.00%	0.00%	13.13%	8.96%
Proportion of total comfirmed	43.48%	72.00%	20.00%	38.24%	43.78%

Abbreviation: AAs, autosomal abnormalities; CNV, copy number variation; CNV-M, copy number variation mosaicism; MA, maternal age; PA, paternal age; SC-CNV, sex chromosome copy number variation; SC-M, sex chromosome mosaicism; T13, trisomy 13; T15-M, trisomy 15 mosaicism; among other abbreviations, trisomy is abbreviated to T and no more redundant description.

We conducted a statistical analysis of true positive rates across different age strata. Among couples with both partners aged ≥35 years, the detection rates for AAs and SCAs were 5.49‰ and 0.61‰, respectively. In contrast, among couples with both partners aged <35 years, the rates were 2.62‰ and 1.31‰, respectively. Detection rates for other chromosomal abnormalities in different age groups are provided in Table 5.

**TABLE 5:** True positive detection rate of NIPT stratified by parental age.

Chromosomal abnormalities	PA ≥ 35 & MA ≥ 35	PA ≥ 35 & MA < 35	PA < 35 & MA ≥ 35	PA < 35 & MA < 35
T13	0	0.22‰	0	0.10‰
T18	1.02‰	0.22‰	0	0.20‰
T21	3.86‰	2.80‰	2.98‰	2.01‰
T15-M	0.20‰	0	0	0
T21-M	0	0	0	0.10‰
T22-M	0	0	0	0.10‰
CNV	0.41‰	0.20‰	0	0
CNV-M	0	0	0	0.10‰
45,X	0	0	0	0.20‰
47,XXX	0	0	0	0.10‰
47,XXY	0	0	0	0.70‰
47,XYY	0.20‰	0.22‰	0	0.20‰
SC-M	0.41‰	0.22‰	0	0
SC-CNV	0	0	0	0.10‰
AAs	5.49‰	3.45‰	2.98‰	2.62‰
SCAs	0.61‰	0.43‰	0	1.31‰

Abbreviation: AAs, autosomal abnormalities; CNV, copy number variation; CNV-M, copy number variation mosaicism; MA, maternal age; PA, paternal age; SC-CNV, sex chromosome copy number variation; SC-M, sex chromosome mosaicism; T13, trisomy 13; T15-M, trisomy 15 mosaicism; among other abbreviations, trisomy is abbreviated to T and no more redundant description.

## 4 Discussion

The clinical application of NIPT technology in detecting aneuploidies such as T13, T18, and T21 in pregnant women has become widely established. Furthermore, the scope of detection is gradually expanding to include additional chromosome abnormalities, such as SCAs and CNVs (Liu et al., 2024). We conducted a retrospective analysis of 19,835 NIPT samples to evaluate the performance of the BGI platform. The PPVs for T21, T18, and T13 were found to be 91.53%, 61.54%, and 14.29%, respectively. Notably, the PPVs for T21 and T18 exceeded those reported by Lu et al. (2024) , while the PPV for T13 was lower than their findings. Additionally, Xu L et al. reported on the performance of Berry Genomics’s assay kit based on the Illumina platform (Xu et al., 2020). Specifically, their results indicated that the PPVs for T21 and T18 (82.52% and 60.71%) were lower than our findings, whereas their result for T13 (25.00%) was higher than ours. All the data and results are shown in Table 6.

**TABLE 6:** Comparison of performance among different detection Kit Companies and Platforms.

Comparison indicators		Our study (BGISEQ)	BGI (BGISEQ)	Berry (Illumina)	Basecare (Proton)
T13/T18/T21	Numbers of cases	19,835	263744^a^	31,515	19,810
	Positive rate	0.51%	0.26%^a^	0.66%	0.82%
	Incidence rate	0.32%	0.15%^a^	0.40%	0.54%
	Total PPV	75.29%	63.88%^a^	77.44%	72.11%
	T13 PPV	14.29%	16.36%^a^	46.67%	25.00%
	T18 PPV	66.67%	43.61%^a^	69.44%	60.71%
	T21 PPV	91.53%	84.14%^a^	84.07%	82.52%
The proportion of AMA		28.51%	8.35%^a^	36.40%	25.30%
Sex chromosomal aneuploidies	Numbers of cases	19,835	222107^b^	31,515	19,810
	Positive rate	0.32%	0.29%^b^	0.71%	0.31%
	Incidence rate	0.07%	0.10%^b^	0.19%	0.14%
	Total PPV	24.56%	48.81%^b^	42.66%	65.12%
	45,X PPV	6.67%	18.14%^b^	25.97%	28.57%
	47,XXX PPV	50.00%	58.73%^b^	65.22%	80.00%
	47,XXY PPV	50.00%	80.29%^b^	75.00%	92.31%
	47,XYY PPV	36.36%	71.19%^b^	83.33%	66.67%
The proportion of AMA		28.51%	11.42%^b^	36.40%	25.30%

Abbreviation: AMA, advanced materal age (materal age is ≥35 years old); PPV, positive predictive value; T13/T18/T21, trisomy 13, 18 and 21; among other abbreviations, trisomy is abbreviated to T and no more redundant description. In the data of BGI, **a** indicates that the data originates from the study conducted by Lu YS, et al. (Lu et al., 2024). **b** means the the data derived from the research reported by Lu Y et al. (Lu et al., 2022). The data of Berry comes from the study reported by Xu L et al. (Xu et al., 2020). Finally, the information pertaining to Basecare comes from the study reported by Xue Y et al. (Xue et al., 2019).

The performance of the Basecare company’s kits based on the Proton platform, as reported by Xue Y et al., indicates that only the PPV for T21 (84.07%) is 7.46% lower than our findings (Xue et al., 2019). In contrast, the PPVs for T18 and T13 are both higher than ours, particularly for T13, which has a PPV of 46.67%. This data is significantly higher than our results and represents the highest value in Table 5. The discrepancy can be attributed to their use of magnetic bead-based enrichment methods for fetal DNA extraction.

In our study, the reported results of composite PPV for T13, T18 and T21 from Xu et al. (2020) and Xue et al. (2019) were found to be higher than those reported by Lu et al. (2024) Consequently, we analyzed the positive rates across the four studies as well as the proportion of advanced maternal age (≥35 years) samples. We discovered that our NIPT positive rate was greater than that in Lu YS et al.'s research (Lu et al., 2024); similar findings were observed in Xu L et al.'s and Xue Y et al.'s reports (Xu et al., 2020; Xue et al., 2019).

Furthermore, the proportion of advanced maternal age participants in our study was 28.51%, significantly higher than that reported by Lu et al. (2024), which stood at only 8.35%. This discrepancy suggests that a higher proportion of women of advanced maternal age may contribute to an increased NIPT-positive rate. This finding is consistent with the conclusion reported by Liu et al. that women of advanced maternal age increases the risk of chromosomal abnormalities in fetuses (Liu et al., 2025; Frick, 2021). The lower percentage pregnant women classified as advanced maternal age in the study (Lu et al., 2024) by Lu YS et al. can be attributed to their data being sourced from a universal NIPT screening program conducted throughout Changsha City.

Currently, NIPT has not been widely adopted as a first-line screening method for detecting chromosome abnormalities during pregnancy in China (Zhao et al., 2023). Typically, it is primarily offered to individuals with abnormal ultrasound soft markers, those classified as advanced maternal age, or patients identified through serum screening who present borderline risks. These groups are more likely to opt for NIPT. Therefore, the proportion of women of advanced maternal age in our study is significantly higher. This finding aligns with the studies conducted by Xu L et al. (36.40%) (Xu et al., 2020) and Xue Y et al. (25.30%) (Xue et al., 2019).

In terms of sex chromosome aneuploidy, our overall PPV was also lower than that reported for the three platforms in Table 5. Among these, Basecare utilizes a magnetic bead-based DNA enrichment method, which showed superior technical performance compared to other platforms. Furthermore, although we also used the BGI platform, our PPV for sex chromosome aneuploidy, was lower than that reported by Yangmei et al. (Lu et al., 2022). This discrepancy may be due to their data being sourced from a government-sponsored public welfare project in Changsha City, where pregnant women had high compliance rates. In contrast, we observed a loss to follow-up of 35 NIPT-positive samples, which likely contributed to the lower PPV in our study.

In recent years, there is considerable discussion regarding the relationship between maternal age and chromosomal abnormalities. However, paternal age has been largely overlooked in prior studies (Wei et al., 2023; Kim et al., 2024; Bonus et al., 2022). To address this gap, we collected paternal age data. As shown in Table 5, when both parents are ≥35 years old, the detection rate of AAs (5.49‰) is significantly higher than that observed when both parents are <35 years old (2.62‰). Among them, the detection rates of T18 (1.02‰) and T21(3.86‰) are relatively high in both parental age is ≥35 years old. However, the detection rate of SCAs in the two groups showed opposite results. We found the higher detection rate of SCAs (1.31‰) in both parental age is <35 years old than in both parental age is ≥35 years old (0.61‰). The results presented in Figure 3C indicate that SCAs are predominantly concentrated in the region where both parents are under the age of 35, except the SC-M. This finding suggests that younger parental age is associated with an increased risk of SCAs compared to advanced parental age groups. Yiwei et al. reported that paternal age <20 years increases the risk of chromosomal disorders in offspring (Fang et al., 2020). However, our cohort lacked samples from fathers <20 years, precluding direct comparison with Yiwei et al.‘s findings.

As shown in Table 4, all cases of 47,XXY (n = 7) were exclusively observed in couples <35 years old with a detection rate of 0.7‰. This novel finding suggests that younger parental age (both partners <35 years) may be a potential risk factor for 47,XXY. Consistent with this, our analysis of sex chromosomal abnormalities revealed that younger parental age was associated with a higher PPV (Table 3). A recent study (Kaltsas et al., 2023) reported by Kaltsas et al. mentioned the same conclusion that the presence of 47,XXY is associated with advanced paternal age, which was inconsistent with our conclusions.

The detection rate of AAs in the advanced paternal age (≥35 years) groups were significantly higher than in the paternal age <35 years group. This suggests a significant association between advanced paternal age and the risk of T13, T18, and T21. Jimbo et al. reported that advanced paternal age is associated with increased rates of chromosomal aneuploidy (Jimbo et al., 2022), which aligns with our findings. Some studies have indicated that paternal age is not associated with the risk of chromosomal disorders (Dviri et al., 2021). However, the study conducted by Sánchez-Pavón et al. revealed that paternal age is a significant risk factor for T21 (Sánchez-Pavón et al., 2022). Whether paternal age is a risk factor for autosomal abnormalities remains inconclusive, which necessitates further research and data to clarify this association. Our results suggest a potential influence of paternal age on the incidence of T21 (2.80‰ VS 2.01‰), but further data collection is required to validate this conclusion.

Notably, all three cases of SC-M were associated with advanced paternal age. Reich et al. found that the rate of SC-M was significantly associated with maternal age, but did not address the potential influence of paternal age (Reich et al., 2020). Given the low incidence of SC-M and the lack of prior studies linking mosaicism to parental age, we hypothesize a potential association that requires validation through larger cohorts.

In China, the age difference between couples is typically small. As illustrated in Figure 3D, linear regression analysis of couples’ ages revealed a strong positive correlation (*R* ^2^ = 0.77). This indicates that advanced maternal age is strongly associated with advanced paternal age. Therefore, when analyzing the relationship between parental age and the incidence of chromosomal abnormalities, it is essential to consider both maternal and paternal ages. The previously reported impact of maternal age on chromosomal abnormalities may result from the combined effect of both parents’ ages.

In summary, we conducted a comprehensive analysis of 5-year NIPT data from our laboratory to assess the performance of the BGI platform and explore correlations between parental age and chromosomal abnormalities in offspring. Our findings demonstrate a significant association between parental age and the incidence of chromosomal anomalies. Specifically, advanced parental age may increase the risk of AAs, whereas younger parental age may elevate the risk of SCAs. These results provide novel insights into clinical strategies for preventing chromosomal abnormalities. The generalizability of our findings may be limited by the sample size, and we did not systematically collect data on the consanguinity status among the participating parents. Consequently, future research should aim to collect more data to strengthen the evidence base for our conclusions.

## Data Availability

The NIPT and diagnostic outcome data supporting the findings of this study have been deposited in Figshare (https://figshare.com) under DOI 10.6084/m9.figshare.28980005. The raw data will be made available by the authors to qualified researchers upon reasonable request. Requests for data access should be directed to the corresponding author via email at 45321874@qq.com.
